# Effect of treatment with epoetin beta on short-term tumour progression and survival in anaemic patients with cancer: a meta-analysis

**DOI:** 10.1038/sj.bjc.6603481

**Published:** 2006-11-21

**Authors:** M Aapro, B Coiffier, J Dunst, A Österborg, H U Burger

**Affiliations:** 1 Institut Multidisciplinaire d'Oncologie, Clinique de Genolier, 1, route du Muids, CH-1272 Genolier, Switzerland; 2 Centre Hospitalier Lyon-Sud, Service d'Hematologie, Chemin du Grand Revoyet, 69495 Pierre-Benite Cedex, Lyon, France; 3 Martin-Luther-University Halle-Wittenberg, Dryanderstrasse 4, D-06097 Halle, Germany; 4 Departments of Oncology and Hematology, Karolinska University Hospital, SE-171 76, Stockholm, Sweden; 5 F Hoffmann-La Roche Ltd, CH-4070 Basel, Switzerland

**Keywords:** anaemia, epoetin beta, survival

## Abstract

To assess the early effect of epoetin beta on survival and tumour progression in anaemic patients with cancer, data were pooled from nine randomised clinical trials comparing epoetin beta with placebo or standard care. Studies were not primarily designed to assess these end points. Follow-up was for treatment duration plus 4 weeks following therapy completion. All adverse events (AEs) were retrospectively reviewed blinded, for progression. Thromboembolic events were also assessed. Data analysis involved standard statistical tests. Overall, 1413 patients were included (epoetin beta, *n*=800; control, *n*=613; 56% haematological, and 44% solid). Median initial epoetin beta dose was 30 000 IU/week. Overall survival during months 0–6 was similar with epoetin beta and control (0.31 *vs* 0.32 deaths/patient-year). No increased mortality risk was seen with epoetin beta (relative risk (RR) 0.97, 95% CI: 0.69, 1.36; *P*=0.87). There was a significantly reduced risk of rapidly progressive disease for epoetin beta (RR 0.78, 95% CI: 0.62, 0.99; *P*=0.042). Epoetin beta was associated with a slightly higher frequency of thromboembolic events *vs* control (5.9% *vs* 4.2% of patients) but thromboembolic-related mortality was identical in both groups (1.1%). Epoetin beta provided a slight beneficial effect on tumour progression and did not impact on early survival or thromboembolic-related mortality.

Anaemia is commonly seen in patients with cancer, and may result from malignant disease itself, or from anticancer treatment. Although the prevalence of anaemia is influenced by both the type of malignancy and the type of therapy, an adverse effect on the quality of life (QoL) of patients may result as a consequence of a variety of symptoms including fatigue, reduced physical capacity, and impaired cognitive function (Cella, 1998; Caro *et al*, 2001). In addition, anaemia is also associated with adverse outcomes in cancer, with individual studies showing shortened survival in patients with a variety of malignancies including cancers of the lung, cervix, head and neck, and prostate cancer, as well as lymphoma and multiple myeloma (Caro *et al*, 2001).

This association between impairment of clinical outcomes and anaemia in patients with cancer has led to the suggestion that prognosis could be improved if anaemia is corrected. Erythropoietic agents have been shown to increase haemoglobin (Hb) levels and reduce transfusion requirements in patients with cancer (Littlewood *et al*, 2001; Österborg *et al*, 2002; Vansteenkiste *et al*, 2002; Boogaerts *et al*, 2003; Bohlius *et al*, 2004). Treatment with epoetin also alleviates fatigue and other symptoms of anaemia and produces significant improvements in QoL compared with placebo or standard transfusion therapy (Österborg *et al*, 2002; Boogaerts *et al*, 2003).

It has been suggested that epoetin therapy may be associated with delayed tumour progression and improved survival in patients undergoing cancer therapy. Preclinical data have indicated that administration of epoetin can enhance response to therapy and delay tumour progression (Mittelman *et al*, 2001; Thews *et al*, 2001; Stuben *et al*, 2003), whereas early clinical studies have suggested a potential survival benefit associated with epoetin in patients receiving radiotherapy and/or chemotherapy (Antonadou *et al*, 2001; Glaser *et al*, 2001; Littlewood *et al*, 2001). This hypothesis is supported by results of a meta-analysis of randomised controlled trials that reported a trend towards increased survival in patients treated with erythropoietic agents (Bohlius *et al*, 2005).

In contrast, an expansion of the above meta-analysis and two recent studies with survival as the primary end point showed no such effect and have even suggested that epoetin therapy might impair survival (Henke *et al*, 2003; Leyland-Jones *et al*, 2005; Bohlius *et al*, 2006). The robustness of these findings has been questioned because of methodological limitations, including, treatment to Hb targets outside of guideline recommendations, use of epoetin beta at twice the recommended dose, protocol violations, and baseline imbalances favouring the placebo group. Nevertheless, contrary to much positive evidence, these studies have fostered the perception that epoetin may have a negative impact on survival and focussed debate on this important question. To help address this question, a meta-analysis of individual patient data from nine controlled clinical studies of epoetin beta was conducted to further investigate any effect on disease progression and survival in patients with cancer.

## MATERIALS AND METHODS

This meta-analysis of individual patient data was designed to evaluate differences between epoetin beta and control (placebo or standard care) with regard to overall survival and malignancy progression during and up to 28 days after therapy. Differences in incidence of thromboembolic events were also assessed.

Eligible studies included all randomised, controlled studies of epoetin beta in patients with cancer undergoing chemotherapy (seven studies) or surgery (two studies) conducted by the drug sponsor (F Hoffmann-La Roche or Boehringer Mannheim). A primary objective was to provide evidence of any effect of epoetin beta on tumour progression or survival. Given the limitations of the Henke *et al* (2003) study design, this radiotherapy study was excluded from the meta-analysis. A further study that evaluated the efficacy of epoetin beta once weekly compared with three times weekly was also excluded because of the lack of a control arm (Cazzola *et al*, 2003). Included studies are summarised in Table 1.

None of the included studies were primarily designed to assess survival and follow-up duration was the standard 4 weeks used to assess AEs. Deaths reported beyond this period were not included as follow-up data were not consistently collected after this period across the studies. Long-term, 12-month follow-up data were available from one study. These longer-term data were excluded from the analysis for reasons of consistency and have been reported elsewhere (Österborg *et al*, 2005). Patients were censored for survival at 4 weeks after the last entry in the administration record. Although tumour status was not assessed in most of these trials, details of malignancy progression were reported as AEs, and these were therefore analysed retrospectively with reviewers blinded to treatment assignment. Patients without events were censored as for survival. Adverse event reports were also reviewed for thromboembolic episodes, with events being classified according to a prospectively designed scheme formulated to standardise the terms used by the different investigating groups across the studies. AEs, for example, hypertension and headache, were routinely monitored in individual studies but were not an objective of the meta-analysis and are therefore not reported. In contrast to studies of epoetin in patients with renal impairment, pure red cell aplasia (PRCA) is a rare event in patients in oncology studies. Therefore, PRCA was not an objective of this meta-analysis and no patient developed PRCA in this meta-analysis population. The incidence of these events is typically low and reported elsewhere (NeoRecormon® SmPC, 2006).

In the study by Oberhoff *et al* (1998), crossover from standard therapy to epoetin beta was permitted, and patients were censored at the time of crossover for the purposes of the present analysis. All patients who received at least one dose of study medication were included in this analysis.

### Statistical analyses

Data on all randomised patients were included in the analyses. Patients were analysed as treated, with five patients randomised to control receiving epoetin beta and three patients in the epoetin beta group receiving no treatment.

Overall and progression-free survival were analysed by Kaplan–Meier estimates, log-rank testing, and Cox regression analysis (the last two not stratified by study). Thromboembolic events were summarised in terms of crude rates independent of onset. Differences in duration of survival and time to tumour progression (time between start of epoetin/control therapy, or baseline visit, and the time of event) were tested using log-rank tests. Patients without an event were censored 28 days after last dose or final visit.

A sensitivity analysis for overall and progression-free survival that excluded studies outside of the normal clinical usage of epoetin beta (two studies in patients undergoing pre-operative cancer surgery and one in patients with acute myeloid leukaemia (AML)) was also performed.

## RESULTS

A total of 1413 patients were included in this analysis (epoetin beta, *n*=800; control, *n*=613). Of these, 56% had haematological malignancies and 44% had solid tumours (Table 2). Five patients originally classified as ‘other’ were later diagnosed with Hodgkin's lymphoma. Most patients with solid tumours had primary malignancies of the breast, colon/rectum, or ovary. Among patients with haematological tumours, 56% had non-Hodgkin's lymphoma, and 42% had multiple myeloma. There were no significant differences in tumour type between treatment groups, with the exception of a slightly higher proportion of patients with ovarian cancer in the epoetin beta group because of the three-arm design used in the study by ten Bokkel Huinink *et al* (1998). Data on tumour stage at baseline was not available for all patients as tumour progression was not a predefined objective in any of the studies. However, information on tumour stage was available for approximately 75% of patients with solid tumours and 80% of patients with haematological malignancies. There was no obvious difference in tumour staging between treatment and control groups.

Median initial weekly epoetin beta dose was 30 000 IU (range 1143–90 000 IU). Mean baseline Hb level was 9.9 g dl^−1^ in both treatment groups whereas mean maximum Hb during treatment was 12.6 g dl^−1^ with epoetin beta and 11.6 g dl^−1^ with control. Mean baseline-adjusted Hb area under the curve was 1.01 g dl^−1^ with epoetin beta compared with 0.16 g dl^−1^ with control, indicating an overall Hb difference of approximately 1.0 g dl^−1^ during treatment.

Duration of follow-up was generally similar in both groups, being limited to treatment duration plus a standard 4-week period. However, more patients were available for follow-up from 4–6 months in the epoetin beta group compared with control, which may have introduced a slight bias favouring control in subsequent analyses.

### Survival

**Figure 1:**
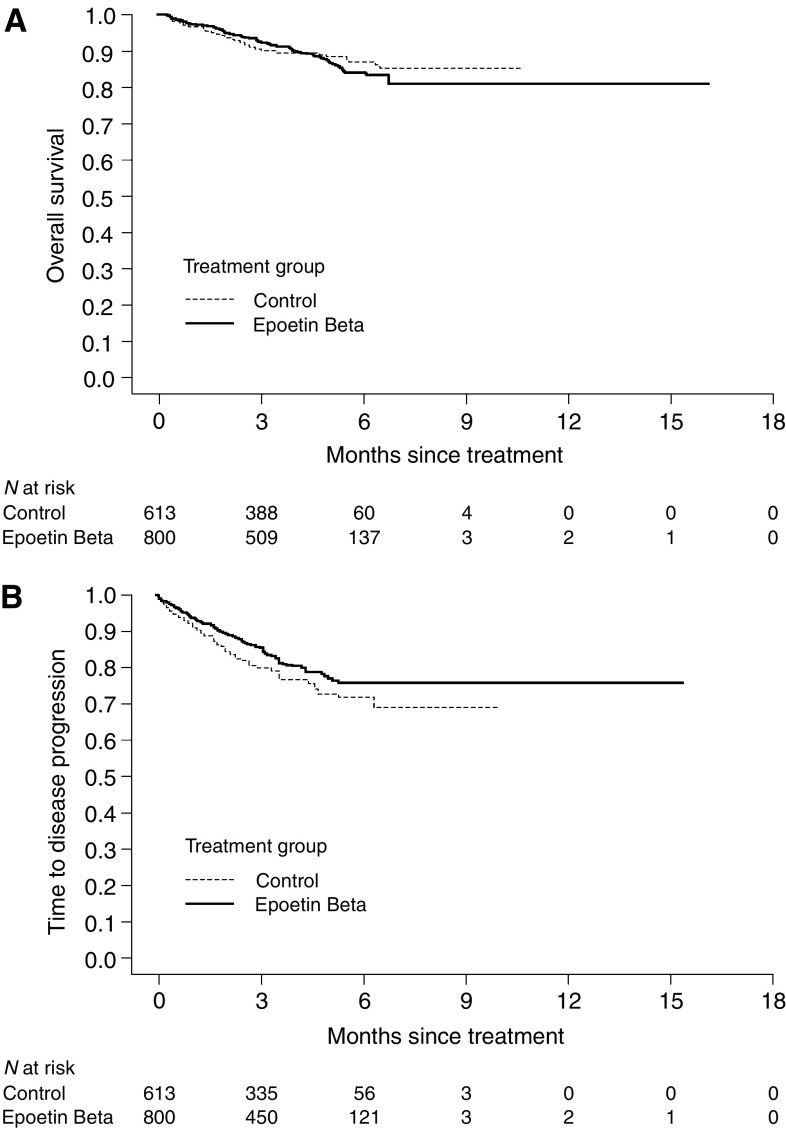
Kaplan–Meier curves of (**A**) overall survival and (**B**) time to progression.

**Figure 2:**
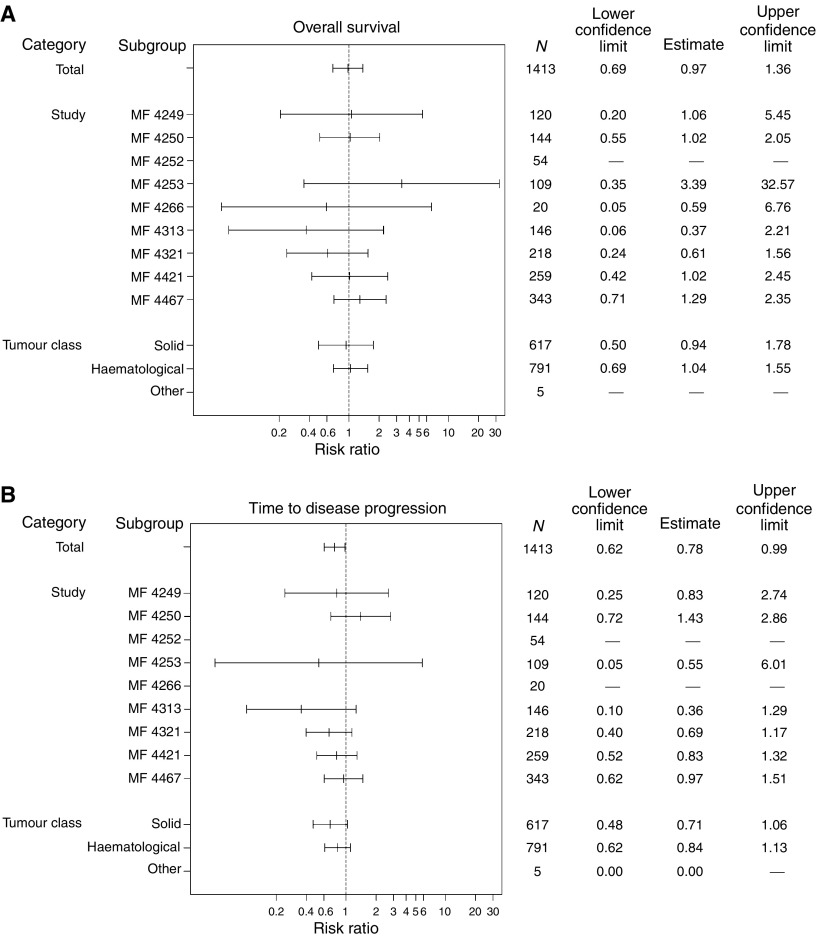
Summary of HRs of (**A**) overall survival and (**B**) time to progression.

There was no significant difference between epoetin beta and control in terms of overall survival during the observation period. The death rate was similar with epoetin beta compared with control (0.31 *vs* 0.32 deaths/patient-year). Kaplan–Meier analysis showed no relevant difference between epoetin beta and control, with respective event rates of 10.0 and 9.5% and an overall hazard ratio (HR) of 0.97 (95% CI 0.69, 1.36; log-rank, *P*=0.87) (Figures 1A and 2A, respectively).

In patients with solid tumours, the death rate was slightly lower with epoetin beta compared with control (0.21 *vs* 0.24 deaths/patient-year), whereas in patients with haematological malignancies the death rate was 0.39 with epoetin beta compared with 0.37 with control. Kaplan–Meier and Cox regression analysis of results by tumour type showed no difference between groups in risk of death for either haematological or solid tumours (Table 3).

Multivariate Cox regression analysis of survival adjusted for the prognostic factors age, gender, tumour type (solid *vs* haematological), and baseline Hb level showed no change in the treatment effect estimates for epoetin beta (adjusted HRs ranging from 0.97 (95% CI 0.69, 1.36) to 1.00 (95% CI 0.71, 1.41). The result of the Cox regression analysis stratified by study was consistent with the primary unstratified analysis (HR 1.04, *P*=0.84).

### Tumour progression

Although there were no apparent clinically significant differences between treatment and control groups in the number of tumour progressions in individual trials, rate of tumour progression was lower with epoetin beta than control in the meta-analysis (0.62 *vs* 0.81 events/patient-year) (Table 3). Kaplan–Meier analysis also showed a reduced risk of progression among patients treated with epoetin beta (HR 0.78, 95% CI 0.62, 0.99; log-rank test, *P*=0.042) (Figures 1B and 2B, respectively).

Subgroup analyses of patients with solid tumours and haematological malignancies were consistent with the overall population (Table 3). Relative risk (RR) for tumour progression with epoetin beta compared with control was 0.71 (95% CI 0.48, 1.06) in patients with solid tumours and 0.84 (95% CI 0.62, 1.13) in patients with haematological malignancies.

As with overall survival, multivariate regression analysis to adjust for prognostic factors (age, gender, tumour type, and baseline Hb level) did not alter the treatment effect estimates (adjusted HR from 0.78 (95% CI 0.62, 0.99) to 0.81 (95% CI 0.64, 1.03)). The result of the Cox regression analysis stratified by study was consistent with the primary finding (HR 0.85, *P*=0.19).

### Exclusion of studies outside normal approved clinical usage

A sensitivity analysis was performed in a subset of patients (epoetin beta, *n*=710; control, *n*=520) that excluded the three studies outside of the normal clinical usage of epoetin beta (two studies in patients with pre-operative cancer surgery (Österborg *et al*, 1996; Rau *et al*, 1998) and one in patients with AML (data on file)). A further study in anaemic patients with head and neck cancer receiving radiotherapy (Henke *et al*, 2003), was also excluded as more than 80% of patients were treated to a Hb level >14 g dl^−1^. Overall results for both survival (HR 0.93, 95% CI 0.66, 1.33; *P*=0.70) and time to progression (HR 0.79, 95% CI 0.62, 1.01; *P*=0.058) were consistent with those for the whole population.

### Thromboembolic events

There was a small excess of thromboembolic events in the epoetin beta group (5.9 *vs* 4.2% of patients with at least one event), which was largely accounted for by reports of thrombosis, deep vein thrombosis, and pulmonary embolism. The proportion of patients who died as a result of thromboembolism was the same (1.1%) in each group. Similar results were obtained when results were analysed by tumour type.

There was a slightly higher incidence of thromboembolism in terms of events per patient-year with epoetin beta over control in most of the studies when considered separately, as well as in this meta-analysis. Incidences were 0.19 events per patient-year in the epoetin beta group and 0.14 in the control group. The RR was slightly lower when analysing events per patient-year than when using crude rates (1.30 for events per patient-year and 1.40 for crude rates). Therefore, differences in the observation time may have contributed to the differences in crude rates.

There were no apparent major differences between studies or between patients with solid tumours compared with haematological malignancies with regard to frequency of thromboembolic events.

## DISCUSSION

The results of the present meta-analysis show no evidence that treatment with epoetin beta impairs survival or promotes tumour progression in patients with cancer, at least during the period of observation.

These data are concordant with evidence from some preclinical studies that suggest that epoetin may improve cyto- and radio-sensitivity and impair progression of various tumours. Correction of anaemia by epoetin has been reported to improve cyclophosphamide cytotoxicity in a rat model (Thews *et al*, 2001) and to restore radiosensitivity of experimental human tumours in nude mice (Stuben *et al*, 2003). Also, one study using a murine myeloma model reported that epoetin induced tumour regression and antitumour immune responses (Mittelman *et al*, 2001). However, other preclinical data, primarily obtained from cell lines, have suggested that epoetin may diminish the effects of cytostatic agents or promote tumour cell growth *in vitro* (Acs *et al*, 2003; Farrell and Lee, 2004).

Some clinical studies have suggested reduced tumour progression and increased survival in anaemic patients with cancer treated with epoetin. In a non-randomised study of 191 patients undergoing neoadjuvant chemoradiotherapy and resection for squamous cell carcinoma of the oral cavity or oropharynx, treatment with epoetin was associated with significantly better local control and survival compared with an untreated historical control group (Glaser *et al*, 2001). Similarly, in a preliminary report, treatment with epoetin improved tumour control and survival in a randomised controlled trial of 385 patients with various pelvic malignancies receiving radiotherapy (Antonadou *et al*, 2001). A nonsignificant trend towards a survival benefit with epoetin has also been suggested by the results of a randomised, double-blind, placebo-controlled trial of 375 patients with solid or non-myeloid haematological malignancies receiving non-platinum-based chemotherapy (Littlewood *et al*, 2001). In addition, a recent meta-analysis by Bohlius *et al* (2005) of randomised controlled trials in patients with cancer also reported a trend towards improved survival with epoetin (HR 0.84, 95% CI 0.69–1.02, *n*=2805). Although subsequent expansion of this analysis showed a shift towards increased mortality risk and increased risk from thromboembolic events, this was suggested by the authors as being possibly due to methodological limitations such as baseline imbalances (Bohlius *et al*, 2006). One prospective study investigating the role of dose-dense chemotherapy in patients with early breast cancer has demonstrated that epoetin alfa had no adverse influence on survival (Möbus *et al*, 2004). A similarly neutral effect on survival was also reported in a meta-analysis of four randomised, double-blind, placebo-controlled studies of darbepoetin alfa in patients with a variety of tumour types (Hedenus *et al*, 2005).

In contrast, two prospective, randomised studies in which survival was a primary end point have been less positive, and have even suggested that therapy with epoetin could have a detrimental effect on survival (Henke *et al*, 2003; Leyland-Jones *et al*, 2005). The study by Henke *et al* (2003) was a double-blind study in 351 patients with carcinoma of the oral cavity, oropharynx, hypopharynx, or larynx treated with curative radiotherapy. In this study, epoetin beta was reported to increase Hb levels relative to placebo. However, there was also a reduction in both locoregional progression-free survival (adjusted RR 1.62; 95% CI 1.22, 2.14; *P*<0.001) and overall survival (adjusted RR of death 1.39; 95% CI 1.05, 1.84; *P*=0.02) relative to placebo. The study by Leyland-Jones *et al* (2005), in which patients receiving first-line chemotherapy for metastatic breast cancer were treated with epoetin alfa for the prevention of anaemia, was terminated early because of a significant (*P*=0.01) difference in 12-month survival between patients in the epoetin alfa (70%) and placebo (76%) groups (Leyland-Jones *et al*, 2005).

However, these two studies need to be interpreted with caution, as baseline imbalances in prognostic factors favoured placebo in both. Other limitations of these two studies have also been highlighted (Dunst, 2004; Leyland-Jones and Mahmud, 2004; Vaupel and Mayer, 2004). Moreover, it should be noted that both trials were investigational in nature and both used epoetin outside of its currently approved indications (in predominantly mild or non-anaemic patients, many of whom attained higher than recommended Hb levels with epoetin therapy). These observations, along with reports that the apparent negative effect of epoetin beta observed in the Henke *et al* (2003) study simply reflects over-treatment (Vaupel and Mayer 2004), led to exclusion of the Henke *et al* study (2003) from this meta-analysis.

This meta-analysis takes into account epoetin beta studies not included in the previous meta-analysis of epoetin randomised controlled trials (Bohlius *et al*, 2005). The findings, together with evidence from other studies (Food and Drug Administration, 2004) suggest there is no indication of an increase in early disease progression and that epoetin does not impair survival in patients with anaemia when used as currently approved. Furthermore, the results of the present study indicate a trend towards a reduced rate of tumour progression with epoetin beta treatment.

Long-term follow-up data from one of the studies included in this analysis provide further evidence that epoetin beta has a neutral effect on survival. In this randomised, double-blind trial of severely anaemic patients with lymphoproliferative malignancy, median survival was 17 months with epoetin beta and 18 months with placebo and Kaplan–Meier curves for survival were similar for both treatment groups (Österborg *et al*, 2005).

Cancer and its treatment are known predictors of risk for thromboembolism, with absolute risk depending on tumour type, stage, and extent of cancer and treatment, and on other factors such as age, surgery, immobilisation, and comorbid features (Lee and Levine, 2003). Treatment with epoetin has been associated with occasional reports of thromboembolic events and this is reflected in the product label. The present meta-analysis, as well as that by Bohlius *et al* (2005), showed a marginal increase in incidence of thromboembolism in patients receiving epoetin. Importantly, in our analysis, epoetin was not associated with any increase in the proportion of patients with thromboembolic events leading to death.

This meta-analysis of nine controlled trials, which represents all randomised, controlled trials of epoetin beta within its current indication, shows no evidence of a negative effect on survival or thromboembolic-related mortality of epoetin beta in patients with cancer. Moreover, these data show a small but statistically significant benefit in slowing tumour progression compared with placebo or standard transfusion therapy. Within its licensed indication, our results indicate that epoetin beta is a safe treatment of anaemia for patients with cancer.

## Figures and Tables

**Table 1:** Main features of randomised clinical trials of epoetin beta in patients with cancer

**Study**	**Design and no. of patients (epoetin beta/control)**	**Diagnosis**	**Epoetin beta dosage and duration of therapy**	**Control**	**Cancer treatment**
ten Bokkel Huinink *et al* (1998)	o, pg *n*=83/37	Ovarian cancer, Hb <13 g/dl	150 or 300 IU/kg 3 × week × 6 months	Standard therapy	Chemotherapy
Österborg *et al*, (1996)	o, pg *n*=95/49	MM, NHL, CLL; transfusion-dependent, Hb<10 g/dl	2000–10 000 IU/day titrated or 10 000 IU/day fixed dosage × 24 weeks	Standard therapy	Chemotherapy
Rau *et al* (1998)	db, pc, and pg *n*=28/26	Resectable rectal cancer; Hb⩾12.5 g/dl (men), ⩾12 g/dl (women)	200 IU/kg daily × 11 days	Placebo	Surgery
Kettelhack *et al* (1998)	db, pc *n*=52/57	Colorectal cancer suitable for hemicolectomy, Hb >8.5–13.5 g/dl	20 000 IU/day × 10–15 days	Placebo	Surgery
Data on file (Study MF4266)	o, pg *n*=10/10	AML	10 000 IU/day, then weekly or twice weekly × ⩽30 weeks	Standard therapy	Chemotherapy
Cazzola *et al* (1995)	o, pg *n*=117/29	MM, NHL, CLL; transfusion-independent, Hb⩽11 g/dl	1000, 2000, 5000, or 10 000 IU/day × 8 weeks	Standard therapy	Chemotherapy
Oberhoff *et al* (1998)	pg *n*=114/104	Solid organ tumours, Hb ⩽11 g/dl	5000 IU/day × 12–24 weeks	Standard therapy	Chemotherapy
Boogaerts *et al* (2003)	o, pg *n*=131/128	Malignant disease, Hb⩽11 g/dl	150 IU/kg 3 × week adjusted for Hb response × 12 weeks	Standard therapy	Chemotherapy
Österborg *et al* (2002)	pc, db, and pg *n*=170/173	MM, NHL, CLL; transfusion-dependent and epo-deficient, Hb⩽10 g/dl	150 IU/kg 3 × week adjusted for Hb response × 16 weeks	Placebo	Chemotherapy

AML, acute myeloid leukaemia; CLL, chronic lymphocytic leukaemia; db, double-blind; Hb, haemoglobin; MM, multiple myeloma; NHL, non-Hodgkin's lymphoma; o, open design; pc, placebo-controlled; pg, parallel group.

Patients had anaemia unless stated otherwise, and standard therapy consisted of antitumour treatment plus blood transfusion as required.

**Table 2:** Baseline characteristics of pooled study populations

**Parameter**	**Control (*n*=613)**	**Epoetin beta (*n*=800)**
Gender (% male)	40	40
		
*Race*		
*N*	481	625
Caucasian	469 (98%)	612 (98%)
Other	12 (2%)	13 (2%)
		
Mean age in years (range)	60.8 (19–91)	61.1 (20–87)
		
*Mean weight in kg (range)*	67.3 (40.0–112.0)	66.8 (35.0–118.0)
*N*	482	663
		
*Mean height in cm (range)*	165.7 (140–198)	165.4 (126–190)
* N*	603	800
		
*Tumour type*		
Haematological^a^	331 (54%)	465 (58%)
Solid	282 (46%)	335 (42%)
		
*Haemoglobin (g/dl)*		
*N*	613	798
Mean (range)	9.94 (5.7–16.7)	9.86 (4.2–17.1)
Median	9.80	9.70

Data were collected from all 1413 patients unless stated otherwise.

a Five patients were originally classified under ‘other tumour type’ but were later diagnosed with Hodgkin's lymphoma.

**Table 3:** Kaplan–Meier and Cox regression analysis of survival and time to progression data

	**Control (*n*=613)**			**Epoetin beta (*n*=800)**			
**Patient group**	**Total events**	**Mean patient-years of follow-up**	**Events per patient-year**	**Total events**	**Mean patient-years of follow-up**	**Events per patient-year**	**Hazard ratio (95% CI)**
*Overall survival*							
Total	58	0.30	0.32	80	0.32	0.31	0.97 (0.69–1.36)
							
*Tumour type*							
Solid	17	0.25	0.24	22	0.32	0.21	0.94 (0.50–1.78)
Haematological	41	0.34	0.37	58	0.32	0.39	1.04 (0.69–1.55)
							
*Time to progression*							
Total	133	0.27	0.81	145	0.29	0.62	0.78 (0.62–0.99)
							
*Tumour type*							
Solid	50	0.23	0.78	50	0.30	0.50	0.71 (0.48–1.06)
Haematological	82	0.31	0.81	93	0.29	0.69	0.84 (0.62–1.13)

‘Events’ refers to number of deaths for ‘overall survival’, and to number of malignant disease progressions for ‘time to progression’.
